# Respiratory Pathogens Adopt a Chronic Lifestyle in Response to Bile

**DOI:** 10.1371/journal.pone.0045978

**Published:** 2012-09-26

**Authors:** F. Jerry Reen, David F. Woods, Marlies J. Mooij, Claire Adams, Fergal O'Gara

**Affiliations:** BIOMERIT Research Centre, Department of Microbiology, University College Cork, Cork, Ireland; The Scripps Research Institute and Sorrento Therapeutics, Inc., United States of America

## Abstract

Chronic respiratory infections are a major cause of morbidity and mortality, most particularly in Cystic Fibrosis (CF) patients. The recent finding that gastro-esophageal reflux (GER) frequently occurs in CF patients led us to investigate the impact of bile on the behaviour of *Pseudomonas aeruginosa* and other CF-associated respiratory pathogens. Bile increased biofilm formation, Type Six Secretion, and quorum sensing in *P. aeruginosa*, all of which are associated with the switch from acute to persistent infection. Furthermore, bile negatively influenced Type Three Secretion and swarming motility in *P. aeruginosa*, phenotypes associated with acute infection. Bile also modulated biofilm formation in a range of other CF-associated respiratory pathogens, including *Burkholderia cepacia* and *Staphylococcus aureus*. Therefore, our results suggest that GER-derived bile may be a host determinant contributing to chronic respiratory infection.

## Introduction

Acid- and non-acid reflux has been associated with increased prevalence of respiratory disease, contributing to increased morbidity and reduced quality of life [1], [2], [3], [4], [5], [6]. A strong correlation between gastro-esophageal reflux (GER), pulmonary aspiration, and increased lung damage has been reported for a wide range of pulmonary conditions [7], including advanced lung damage arising from lung transplantation [8], [9], [10], [11], ventilator associated pneumonia [12], Barrett’s esophagus, and esophageal adenocarcinoma [13]. Most significantly, gastro-esophageal reflux (GER) has also been linked to infection and reduced lung function in Cystic Fibrosis (CF) patients [14], [15], [16].

CF is the most common inherited fatal disease in the Caucasian population and chronic respiratory infection remains the leading cause of morbidity and mortality in these patients [17], [18]. CF patients are highly susceptible to microbial colonisation, have reduced lung function, and suffer thick viscous secretions arising from defective transport of ions across the epithelium. *Pseudomonas aeruginosa* is the primary pathogen associated with CF and, in spite of improved antimicrobial therapy, once a patient becomes chronically infected with *P. aeruginosa* (60–80% of children and adults with CF) it is impossible to eradicate [19]. This is largely due to the fact that in order to evade the host’s immune response, pathogens modulate their behaviour during chronic infection by increasing resistance to antibiotics, adapting a biofilm-like mode of growth and reducing toxin production [20]. Thus, *P. aeruginosa* chronic infection of the lungs of CF patients leads to a progressive and eventually fatal decline in lung function [20]. These behavioural changes contrast significantly with those observed during acute infections where *P. aeruginosa* is capable of causing pneumonia, breaking down lung defenses and disseminating in the bloodstream. This requires a battery of acute virulence factors including a Type Three Secretion System (T3SS) and a range of extracellular toxins, potentially leading to death of the patient within hours or days [21], [22].

GER is a common complication in CF patients with 35 to 80% of the patients suffering from this disease [23], [24], [25], [26], [27], [28]. Essentially, GER occurs when the contents of the stomach leak into the esophageal tract causing heartburn and other symptoms. Moreover, the essential daily physiotherapy regimen of CF patients to clear their airways, also leads to increased GER [29]. GER aggravates chronic bronchopulmonary diseases by promoting aspiration or bronchospasm, contributing ultimately to poorly controlled CF and end-stage lung disease [30]. Respiratory insufficiency arising from GER in CF patients generally requires bilateral lung transplant, which carries considerable morbidity and mortality [30]. GER has also been shown to result in neutrophilic airway inflammation in respiratory patients [31]. However, despite the prevalence of GER in CF patients, and the established correlation with increased inflammation and reduced pulmonary function, current medical management therapies to counter reflux are limited and remain less effective than laparoscopic fundoplication surgery (stomach wrap) [32].

Although the mechanism underlying the impact of GER on lung damage remains unknown, recent evidence of aspiration of GER-derived bile into the lungs of respiratory patients has been reported [2], [12], [23], [26], [33]. In the case of CF patients, the incidence of bile aspiration could be as high as 80% [2]. Similarly, D’Ovidio and colleagues detected elevated concentrations of bile salts in the bronchoalveolar lavage (BAL) of 120 post-transplant patients in a cross sectional study, with the highest concentrations found in patients with early onset bronchiolitis obliterans syndrome (BOS) [34]. The authors also found that elevated BAL bile acids were associated with alveolar neutrophilia, interleukin 8 and cultures positive for bacteria and fungus. Interestingly, a correlation between bile aspiration and airway colonisation with *P. aeruginosa* has been reported following lung transplantation [10], [11], [35] and in retrospective studies on CF children [14], [16], [36]. This raised the intriguing question as to whether GER-derived bile may influence the behavior of pulmonary microflora, thus contributing to the increased lung disease associated with GER.

Therefore, this study was designed to investigate the impact of bile on the behaviour of respiratory pathogens. Strikingly, physiologically relevant concentrations of bile were found to induce phenotypes required for persistence and chronic infection while suppressing phenotypes associated with the acute phase of infection in *P. aeruginosa*. This raises the intriguing possibility that GER-derived bile may be a major host determinant in the establishment of chronic infection.

## Materials and Methods

### Bacterial Strains, Plasmids, and Media

Bacterial strains used in this study were as follows; *P. aeruginosa* strains PA14, PAO1 and clinical isolates CF242 and CF194, *Burkholderia cenocepacia* 10743, *Pandorea spuronum* LMG18819, *Staphylococcus aureus* NCDO949, *Acinetobacter baumannii* and *Stenotrophomonas maltophilia* clinical isolates (Table 1). Cultures were routinely grown in Tryptic Soy Broth (TSB) or Luria–Bertani (LB) media at 37°C with shaking at 180 rpm. For analysis of Type Three Secretion promoter activity, cells were grown in LB-NTA media. Where appropriate, antibiotics were added to growth media at the following concentrations: 200 µg/mL carbenicillin, 200 µg/mL streptomycin, 600 µg/mL trimethoprim, and 50 µg/mL tetracycline. Bovine bile (Sigma-Aldrich) and Bacto bile salts (Difco) were added to the media prior to autoclaving at 105°C for 30 min. For biofilm assays, bovine bile was prepared in sterile distilled water and filter sterilised prior to addition to media. All experiments were performed in both the PA14 and PAO1 strains, with the exception of the T6SS and AHL reporter fusion analyses, which were performed in PA14 and PAO1 respectively. The PA14 data is presented in this paper and is representative of both strains.

**Table 1:** Bacterial strains and plasmids used in this study.

Strain	Description	Source
*P. aeruginosa*		
PA14	Wild-type	[77]
PAO1	Wild-type	B.W. Holloway*
CF194	CF clinical isolate	[78]
CF242	CF clinical isolate	[78]
*A. baumannii*	CF clinical isolate	VU?
*S. maltophilia*	CF clinical isolate	VU?
*P. spuronum* LMG18819	CF clinical isolate	E. Caraher#
*S. aureus* NCDO949	Type strain	Shinfield, UK.
*B. cepacia* NCTC10743	Type strain	Collindale, UK.
**Plasmids**		
pLP0996	*pqsA-lacZ* promoter fusionin pLP170	[46]
pMP*rhlI*	*rhlI-lacZ* promoter fusionin pMP220	[47]
pMP*lasI*	*lasI-lacZ* promoter fusionin pMP220	[47]
pCTX*exoU*	*exoU-lux* pminiCTX chromosomal fusion	[64]
pMS*tssA*	*tssA*-*lux* promoter fusionin pMS402	This study

* Monash Unniversity, Clayton, Victoria, Australia.

# Centre of Microbial Host Interactions, ITT Dublin, Tallaght, Dublin 24, Ireland.

? University Medical Center, Amsterdam, The Netherlands.

### Growth Analysis

Overnight cultures of PA14 were inoculated at starting optical density at 600 nm (OD_600 nm_) of 0.02 into 20 ml of fresh (TSB and LB) media in conical flasks, supplemented with increasing concentrations of bile ranging from 0.03–0.3% (w/v). Cultures were grown at 37°C with agitation at 180 rpm and samples were taken over a 24 hr period.

### Semi-solid Motility Assays

Swarming motility assays were performed on semisolid (0.6%) Eiken agar plates as described previously [37]. Bile was added at a final concentration of 0.03–0.3% (w/v) prior to autoclaving at 105°C for 30 min. Sterile toothpicks were used to inoculate colonies from plates, incubated overnight, onto the centre of motility plates. All experiments were performed in triplicate and data presented is representative of at least three independent biological replicates.

### Pellicle Formation and Attachment Assays

Bacterial cultures were incubated overnight and transferred into fresh TSB to an OD_600 nm_ of 0.05 in glass tubes and bile was added at a final concentration of 0.03–0.3% (w/v). Samples were incubated overnight and pellicle formation at the liquid-air interface was evaluated by crystal violet staining after washing to visualise attached cells. Attachment assays were performed in 24-well plates adding 1 ml of OD_600 nm_ 0.05 cultures and incubating at 37°C for 18 hr. Plates were washed in water and attached cells were quantified using a crystal violet (0.1% w/v) stain. Artificial sputum medium (ASM) was prepared as described by Sriramulu and colleagues [38]. ASM media (1 ml) was inoculated to OD_600 nm_ of 0.05 of an overnight culture of *P. aeruginosa* PA14 in 24-well cell-culture plates. Plates were then incubated for 72 hr at 37°C and 150 rpm and microcolony formation was visualised as described previously [39]. All experiments were performed in triplicate and data presented is representative of at least three independent biological replicates.

### Alkylquinolone and AHL Extraction

For extraction of alkylquinolones, overnight cultures of *P. aeruginosa* were diluted to OD_600 nm_ 1.0 in fresh LB medium [40]. A 250 µl aliquot was transferred into 25 ml of fresh LB media with and without 0.03–0.3% (w/v) bile in a 250 ml conical flask and incubated at 37°C with agitation. Cell-free supernatants were obtained after 5 and 8 hr growth, and quorum sensing signal molecules were extracted using equal volumes of acidified ethyl acetate (0.01% glacial acetic acid) with the solvent being subsequently removed by rotor evaporation. Extracts were resolubilised in 2 ml of methanol, evaporated under nitrogen gas, and finally resuspended in 1 ml of methanol for further analysis. Extraction of AHLs proceeded as above with the exception that extracts were solubilised in ethyl acetate.

### Thin Layer Chromatography (TLC) Analysis

For analysis of alkylquinolones, extracts were spotted on normal phase silica TLC plates (Merck) (soaked for 30 min in KH_2_PO_4_ and activated at 100°C for 1 hr before use), using capillary tubes [40]. Chromatograms were developed in dichloromethane:methanol 95∶5 and visualised directly under UV light. Synthetic PQS and HHQ were loaded as controls [41].

Extracted AHL signal molecules were analysed on C_18_ reversed-phase TLC plates (Whatman, Clifton, NJ, USA). Aliquots of 20 µl were spotted using capillary tubes and the chromatograms were developed with methanol: water (60∶40, v:v) before air-drying in a fume hood [42]. The TLC plate was then overlaid with a thin film of LB agar (1% w/v) seeded with the AHL reporter strain *Chromobacterium violaceum* CV026 that produces the blue colour violacein in response to AHLs with long *N*-acyl side chains [43]. After incubation of the plate at 30°C for 24 hr, AHLs were located as purple spots on a white background. Alternatively, TLC plates were overlaid with agar seeded with a culture of the reporter bacterium *Serratia marcescens* SP19, which detects short chain AHLs, this time as red pigmented spots on the white TLC plate [44].

### Promoter Fusion Analysis

Overnight cultures of *P. aeruginosa* strains containing relevant promoter fusions described in Table 1 were diluted to OD_600 nm_ 0.02 in 20 ml LB in the presence and absence of bile (0.03–0.3% w/v) and grown at 37°C with shaking at 180 rpm. *β*-Galactosidase activity from promoter-*lacZ* reporter fusions was measured over time as described by Miller [45]. The *pqsA-lacZ* plasmid pLP0996 has been widely used to investigate promoter activity of the *pqsA-E* PQS biosynthetic operon [46]. The AHL promoter fusion plasmids were previously used to assess *rhlI* and *lasI* promoter activity [47]. Bioluminescence from promoter-*lux* reporter fusions was measured on a GENios FluorX4 luminescence microplate reader (Tecan). Promoter activity of the *exoU* effector has routinely been used as an indicator of T3SS production and the pCTX*exoU* plasmid has been used to this effect in previous studies [48].

### RNA Extraction and cDNA Synthesis

*P. aeruginosa* PA14 cells were grown overnight in LB media at 37°C with shaking at 180 rpm. Cells were transferred into LB-NTA medium (supplemented with and without bile 0.3%) at a starting OD_600 nm_ 0.01, were incubated at 37°C with shaking at 180 rpm, and were harvested at OD_600 nm_ 0.8. RNA was stabilised using RNA Protect (QIAGEN) and isolated using an RNeasy Mini kit (QIAGEN). RNA was DNase treated using RQ1 RNase-Free DNase (Promega) and reverse transcribed using AMV Reverse Transcriptase (Promega).

### Real Time RT-PCR Analysis

RealTime PCR was conducted on a Chromo4 Continuous Fluorescence Detector (MJ Research) using FastStart TaqMAN Probe Master and probes #20 and #124 from the Universal ProbeLibrary (UPL, Roche). Oligonucleotide primers TaqProC-F CTTCGAAGCACTGGTGGAG and TaqProC-R TTATTGGCCAAGCTGTTCG were used in conjunction with probe #20, while TaqExoU-F GAGCGAGTCGGTGAACATCT and TaqExoU-R AAGCGAAGGTATGACGCTCT were used in conjunction with probe #124. *ExoU* transcript levels were standardised against the housekeeping gene *proC* for comparison between bile treated and untreated samples.

### Statistical Analysis

Three independent biological replicates were performed for all experiments described in this manuscript. Statistical analysis was performed using two-tailed paired Student’s t-test. Differences were considered significant if the p-value was ≤0.05.

## Results

### Bile Induces *P. aeruginosa* to Adopt Biofilm Mode of Growth

**Figure 1:**
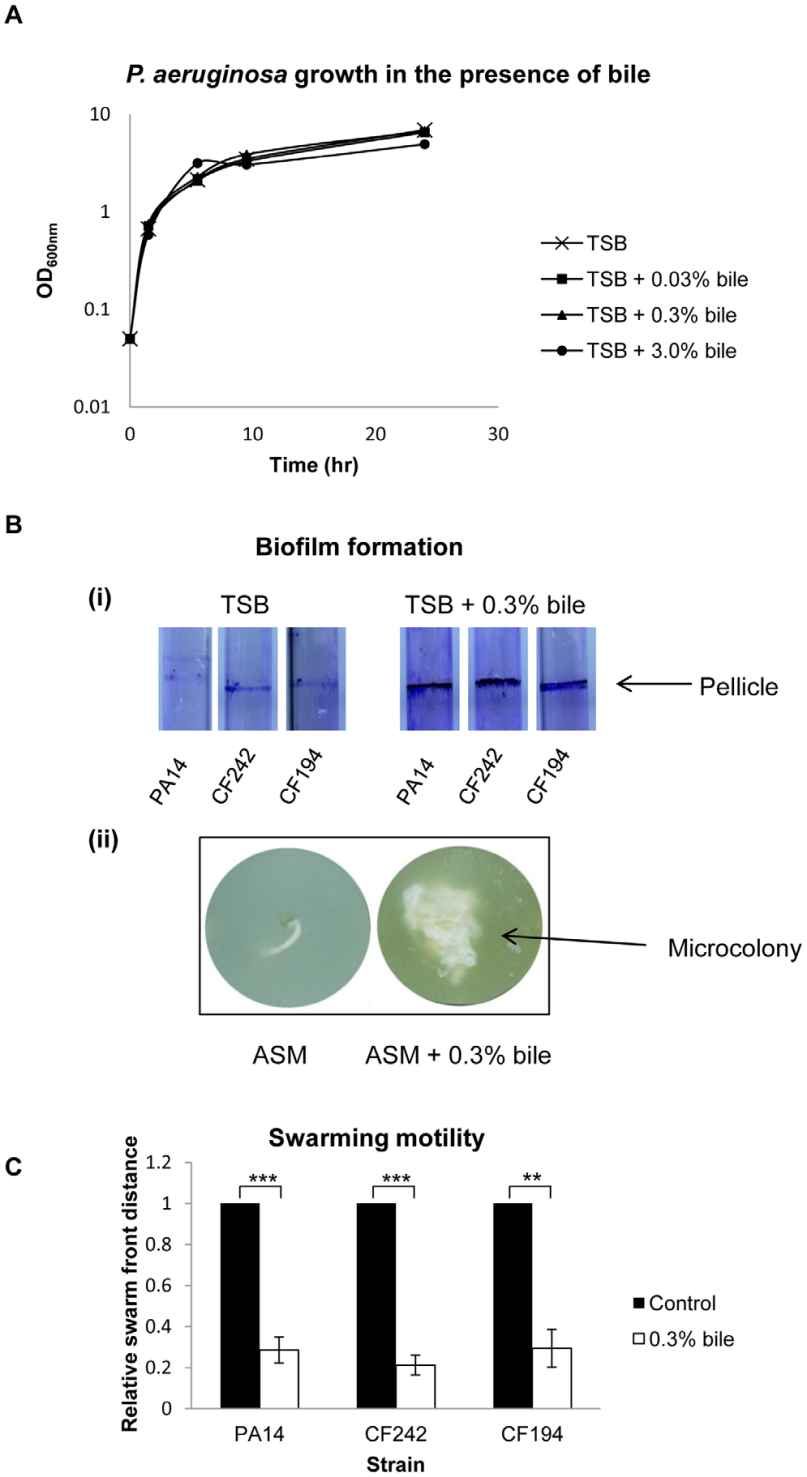
Bile exposure causes *P. aeruginosa* to adopt biofilm mode of growth. (**A**) Growth of *P. aeruginosa* in the presence of increasing concentrations of bile revealed concentrations up to 3% to be subinhibitory to growh of this pathogen. (**B**) (**i**) Crystal violet stain of pellicle formation in glass tubes revealed significant increase in the presence of 0.3% (w/v) bile compared to media alone. This response was maintained in two CF clinical isolates, CF242 and CF194. (**ii**) Microcolony formation in ASM media is significantly increased in the presence of 0.3% bile. (**C**) Swarming is significantly repressed in the presence of 0.3% bile compared to media alone. Swarm distance was measured and compared to control samples. The control was arbitrarily designated as 1 and the data generated in presence of bile was normalised to this value and presented with the standard error of the mean (SEM; p<0.005). All experiments were performed in triplicate and data presented is representative of at least three independent biological replicates.

The emerging correlation between GER-derived bile aspiration and reduced lung function in CF patients raised the intriguing possibility that this host determinant could modulate the virulence of respiratory pathogens. Therefore, the influence of physiologically relevant subinhibitory concentrations of bile on virulence traits associated with *P. aeruginosa* infection was investigated (Fig. 1). Interestingly, while GER derived acid-reflux is typically associated with lowering of the pH of the respiratory tract [24], addition of bile to the media did not change the pH which remained at 6.8+/−0.1. Microbial biofilms, where bacteria form structured communities embedded in an extracellular matrix, are a key feature of chronic infection and in the case of *P. aeruginosa* are often associated with loss of the single polar flagellum [49]. Addition of exogenous bile was found to significantly increase attachment of *P. aeruginosa* to abiotic surfaces compared to controls (Fig. 1Bi & Fig. S1). In particular, pellicle formation at the liquid air interface was increased in the presence of bile, while bile supplemented media alone did not attach to the glass or stain with crystal violet. To further investigate the influence of bile on biofilm formation in *P. aeruginosa*, microcolony formation in artificial sputum media (ASM), which is considered to represent biofilm formation in the CF lung, was assessed. Similar to the increased pellicle formation observed in glass tubes, microcolony formation was also increased upon addition of bile (Fig. 1Bii). Biofilm is inversely associated with swarming motility, which requires both a functional flagellum and the production of rhamnolipid surfactants. Interestingly, bile also significantly repressed swarming motility of *P. aeruginosa* (Fig. 1C & Fig. S1). The swarm distance of cells in the presence of bile was significantly reduced relative to that in the absence of bile. Microscopic visualisation of *P. aeruginosa* cells revealed that bile interfered with the production of flagella possibly underpinning the reduction in motility (data not shown). Interestingly, swarming motility and biofilm/microcolony formation were also significantly affected in the presence of bile salts (data not shown), suggesting that bile acids may be a major factor in triggering this response.

*P. aeruginosa* CF isolates have previously been shown to display strain-specific biofilm formation when compared with non-CF isolates, and several studies have revealed specific adaptation to the CF lung [50], [51]. Therefore, to investigate the clinical significance of the bile response observed in wild-type PA14 and PAO1, both of which were initially isolated from burn wound patients, two additional *P. aeruginosa* clinical isolates obtained from an Irish CF clinic, CF242 and CF194, were assessed for biofilm formation and swarming motility. Biofilm formation was increased while swarming motility was repressed in the *P. aeruginosa* clinical isolates in the presence of bile to the same degree as the reference strains used suggesting that the molecular pathways that transduce the bile response are functional these clinical strains (Fig. 1 and Fig. S1).

### AHL and Alkyl Quinolone (AQ) Quorum Sensing Systems are Triggered in Response to Bile

**Figure 2:**
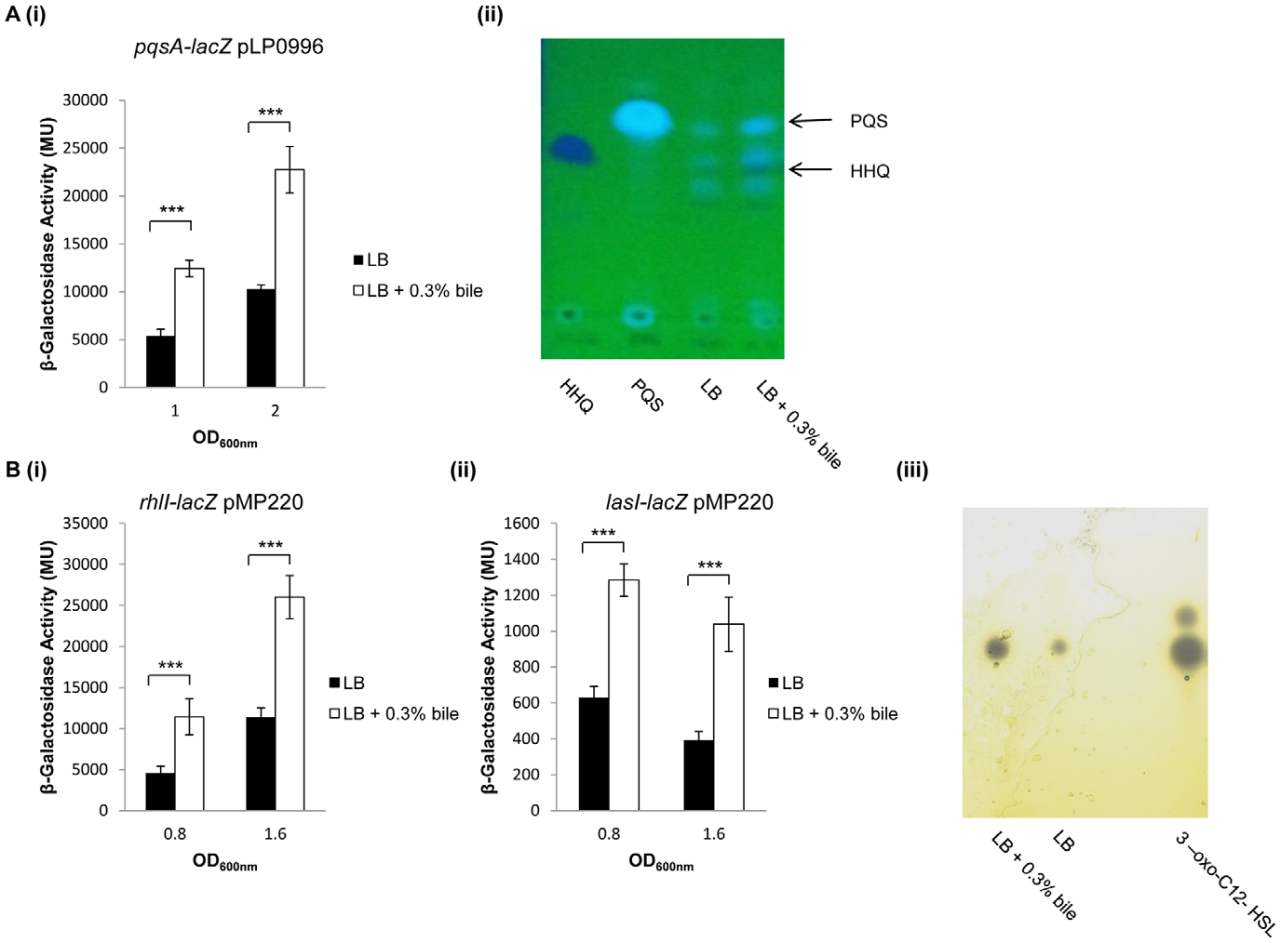
QS is triggered in response to bile. (**A**)(**i**) Promoter activity of the *pqsA-E* biosynthetic operon was significantly increased in the presence of 0.3% bile (p<0.005). (**ii**) TLC analysis of supernatant extracts from cells grown in the presence and absence of 0.3% bile revealed that the increased expression of the *pqsA-E* operon in the presence of bile results in increased production of the HHQ and PQS signal molecules. Synthetic HHQ and PQS were loaded as controls to facilitate identification of the signal molecules in the supernatant extract. (**Bi & ii**) Promoter activity of the *rhlI* and *lasI* AHL QS systems was also significantly increased in the presence of 0.3% bile (p<0.005). As with the PQS system, increased promoter activity was evident in early and late exponential phase growth. (**Biii**) Reversed phase TLC analysis of supernatant extracts taken from bile treated and un-treated PA14 cells grown in LB, and overlaid with soft agar containing the *C. violaceum* CV026 long chain AHL biosensor strain. Increased production of the long chain AHLs can clearly be seen in the treated sample corresponding to the position of the 3-oxo-C12-HSL standard (25 µg). All experiments were performed in triplicate and data presented is representative of at least three independent biological replicates.

The switch from a planktonic to a biofilm mode of growth requires the concerted action of several signalling systems collectively termed quorum sensing (QS) [52]. *P. aeruginosa* encodes three QS systems, two of which (LasI/R and RhlI/R) are classical AHL-based pathways, and the third is a *P. aeruginosa* specific AQ system. Consistent with a role for this system in establishing biofilms in *P. aeruginosa*, bile increased promoter activity of the *pqsA-E* AQ biosynthetic operon (Fig. 2Ai). Expression was increased in early log phase and remained elevated into stationary phase. Confirmation of the inductive effect of bile on the production of the Pseudomonas quinolone signal (PQS), and its biological precursor 2-heptyl-4-quinolone (HHQ), was obtained through TLC analysis of extracts taken from cultures of *P. aeruginosa* grown in the presence/absence of bile (Fig. 2Aii). Both PQS and HHQ were shown to be present at higher concentrations in bile-treated samples, with Rf values consistent with synthetic controls. Interestingly, promoter activity of the *lasI* and *rhlI* signal components of the two AHL QS systems was also increased in the presence of bile (Fig. 2Bi & Bii). Analysis of supernatant extracts on C18 reversed-phase TLC plates overlaid with AHL biosensor strains *C. violaceum* CV026 and *Serratia* SP19 revealed an increase in production of both long and short chain AHL molecules in the presence of bile (Fig. 2Biii). Along with PQS, these AHL systems in *P. aeruginosa* are known to contribute to the biofilm mode of growth, although the role of QS in late stage chronic infections remains unclear arising from the prevalence of *lasR* mutations detected in isolates taken from CF-sputum [53].

### Type Six and Type Three Secretion Systems (T6SS and T3SS) are Inversely Regulated in Response to Bile

**Figure 3:**
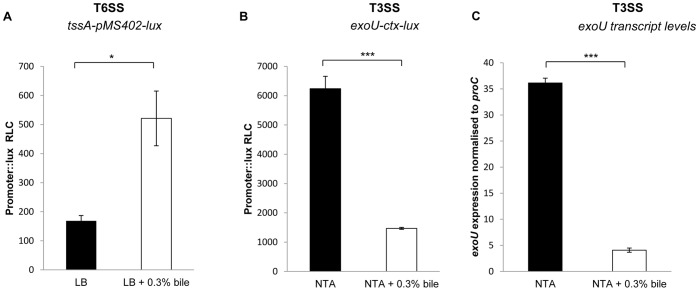
Bile modulates inverse expression of the T6SS and T3SS. (**A**) Promoter activity of the T6SS promoter *tssA*, which has been implicated in chronic infection, was significantly increased in the presence of 0.3% bile (p<0.05). (**B**) Promoter activity of the T3SS effector *exoU*, associated with acute infection, was significantly decreased in NTA medium in the presence of 0.3% bile (p<0.005). Previous studies have reported the inverse relationship between expression of these two systems. (**C**) Real Time RT-PCR expression analysis performed using UPL probes revealed that transcript levels of *exoU* were reduced >8 fold in NTA medium in response to 0.3% bile (p<0.005). All experiments were performed in triplicate and data presented is representative of at least three independent biological replicates.

Microbial secretion systems also play an integral role in the establishment and persistence of respiratory pathogen infections. Therefore, the expression of genetic markers representing two of these systems was studied in the presence of bile. The T6SS Hcp-secretion island (HSI-1) system has previously shown to be required for chronic infection [54]. Promoter activity of the *tssA* gene, the transcriptional origin of a HSI-1 operon, was induced 3-fold (+/−0.8) in response to exogenous bile (Fig. 3A). Conversely, the T3SS is an important weapon during acute infection and is known to be triggered by a range of host-related signals, including reduced Ca^2+^ and limiting oxygen [48]. Addition of bile to *P. aeruginosa* PA14 carrying an *exoU*-promoter fusion revealed that expression of this key T3SS effector toxin was significantly repressed 4-fold (+/−0.9) in T3SS-inducing NTA medium in response to this novel host respiratory signal (Fig. 3B and Fig. S1). Furthermore, Real-Time RT-PCR analysis using the Universal Probe Library (Roche) revealed that transcript levels of the T3SS effector *exoU* were 8.9 fold (+/−0.64) lower in cells grown in the presence of bile, confirmation of the reduced promoter activity identified through promoter fusion analysis (Fig. 3C). Therefore, bile exposure would appear to promote T6SS secretion and repress T3SS secretion in *P. aeruginosa*, a phenotypic switch reflecting a behavioural change towards a chronic lifestyle.

### Bile Modulates Biofilm Formation in other CF-associated Respiratory Pathogens

**Figure 4:**
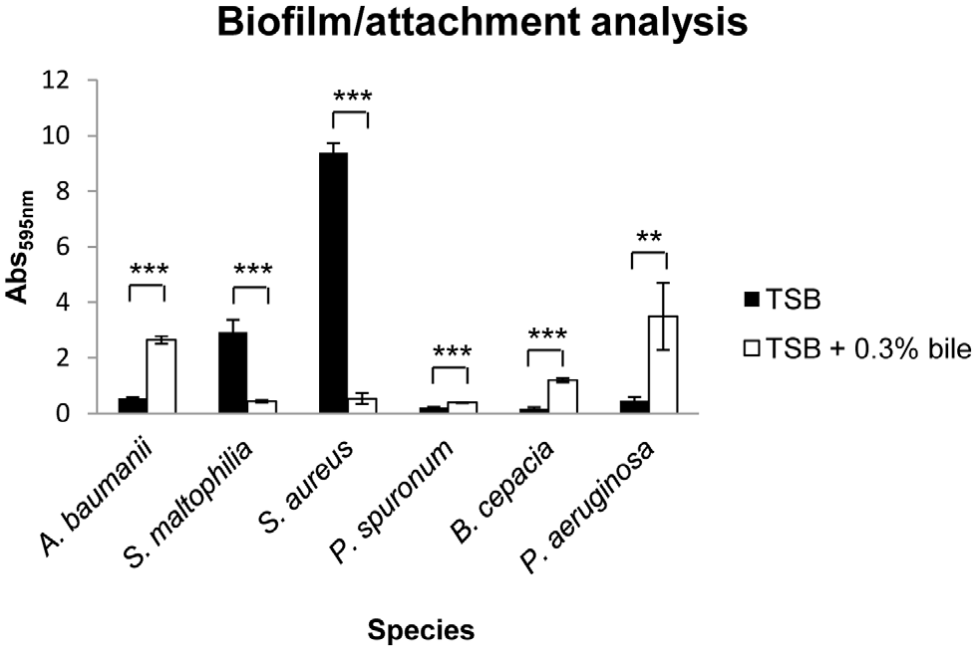
Bile modulates biofilm formation in a broad spectrum of CF-associated pathogens. Addition of subinhibitory concentrations of bile (0.3%) significantly increased biofilm formation in *A. baumanii*, *P. spuronum*, *B. cenocepacia*, and *P. aeruginosa*. Conversely, biofilm formation was significantly reduced in *S. maltophilia* and *S. aureus* at concentrations that are subinhibitory to growth. All experiments were performed in triplicate and data presented is representative of at least three independent biological replicates (**p<0.01, ***p<0.005).

The capacity of bile to influence the behaviour of *P. aeruginosa* towards a chronic biofilm mode of growth *in vitro* led us to investigate the influence of bile on a range of diverse respiratory pathogens that compete or co-exist with *P. aeruginosa* during infection of the CF-lung. Analysis was performed in multi-well plates and each pathogen was assessed in the presence of a range of subinhibitory concentrations of bile. Strikingly, while bile enhanced biofilm formation/attachment in *A. baumanii*, *B. cepacia* and the emerging CF pathogen *P. spuronum*, it strongly repressed this key chronic phenotype in *S. aureus* and *S. maltophilia* (Figs. 4 & S1). The formation of a strong pellicle was particularly evident in *B. cepacia* while the inability of *S. aureus* and *S. maltophilia* to attach to the surface of the plate in the presence of bile was striking. The species-specific response of respiratory pathogens to bile raises the intriguing possibility that this novel host signal could play a major role in the clinical outcome of respiratory infections.

## Discussion

**Figure 5:**
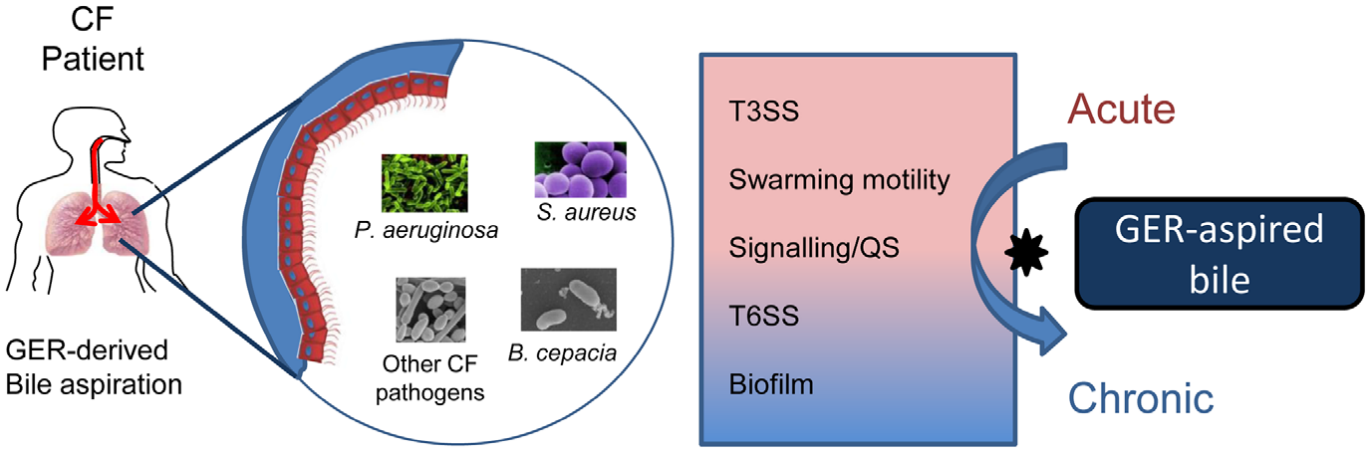
GER-derived bile may influence respiratory pathogen behaviour and biodiversity in the CF pulmonary system. Bile aspiration linked to GER is highly prevalent in CF patients and has been linked to reduced pulmonary function in these patients. As bile is shown to influence key phenotypes associated with chronic infection in *P. aeruginosa*, GER-derived bile may constitute a major host factor in chronic respiratory disease. Bile induces biofilm formation, T6SS, and QS in *P. aeruginosa*, while T3SS and swarming motility are repressed. The transcriptional and phenotypic changes are consistent with a switch towards the chronic lifestyle adopted by *P. aeruginosa* and other respiratory pathogens in the CF lung. Bile also influences biofilm formation in other respiratory pathogens prevalent in CF patients suggesting that GER-derived bile may influence biodiversity in the CF lung.

Physiologically relevant subinhibitory concentrations of bile alter the behaviour of *P. aeruginosa* and other respiratory pathogens, causing them to adopt a chronic lifestyle (Fig. 5). This is highly significant in light of the prevalence of GER-derived bile aspiration in CF patients and may offer an insight into the emerging correlation between GER and airway colonisation by *P. aeruginosa*. While studies have suggested that the increased colonisation by *P. aeruginosa* in patients with GER may arise from aspiration of the bacteria into the lung [14], our data suggests that bile aspiration itself may predispose the pathogens to a chronic and persistent lifestyle. Therefore, there may be an underlying association between the behavioural changes elicited by bile and the increased prevalence of *P. aeruginosa* and perhaps other respiratory pathogens in GER positive patients.

GER-derived bile aspiration may have a significant influence on the behaviour and biodiversity of respiratory pathogens in a range of clinical conditions, potentially an unforseen factor contributing to increased lung damage and morbidity in these patients. Furthermore, the number of patients suffering from GER may be considerably higher than reported in several of these studies given the limitations to detection and clinical diagnosis of this condition [55]. While concentrations as low as 0.03% of complex bile reflect the low micromolar range of bile acids detected in the respiratory tract [2], [56], 0.3% bile represents the physiological range present in the stomach [57]. Therefore, exposure to bile may also have a major influence on the pathogenesis of *P. aeruginosa* outside of the respiratory tract, e.g. in the biliary duct of patients from where it is increasingly being isolated [58], [59]. Interestingly, the influence of bile on respiratory pathogen behaviour would appear be dose dependent. While biofilm formation was significantly influenced in the presence of 0.03% bile, the impact of 0.3% bile on biofilm formation was more pronounced. This dose dependency was further confirmed by the effect of bile on QS promoter activity where 0.03% increased *pqsA*, *rhlI* and *lasI* promoter activity ∼1.3-fold while 0.3% bile increased promoter activity at least 2-fold. In contrast, the influence of 0.03% bile on T3SS and swarming motility was only slightly less potent than 0.3%. Further analysis will be required to determine the full extent of the influence of bile on respiratory pathogen behaviour and global transcriptomic and proteomic profiling will provide a useful insight in this regard.

The finding that bile elicits chronic behaviour in *P. aeruginosa* and other pathogens is of major clinical significance as, following the establishment of chronic infection, eradication of the pathogen is no longer feasible and the therapeutic goals change from attempting to cure the infection to slowing the decline of lung function and improving the patient’s quality of life. However, while insights into regulatory pathways involved in chronic infection have been reported [48], [49], [60], [61], [62], [63], [64], [65], [66], [67], the specific signals that elicit the transition from an acute to a chronic infection remain unknown. Our data suggests that the accumulation of bile and perhaps other novel signals in the respiratory tract and lungs of patients with GER may activate signal neworks and rewire the transcriptional machinery, eliciting the switch towards persistent lifestyle. The increased biofilm formation observed in the presence of bile correlates with increased production of PQS, loss of flagella, and reduced swarming motility. PQS has previously been shown to contribute to *P. aeruginosa* biofilm formation, potentially mediating complex subpopulation interactions [68]. Furthermore, increased production of C4-HSL through the RhlIR system has been shown to result in reduced T3SS, consistent with the data presented here [69]. Potential molecular mechanisms may involve the RetS/GacS/LadS/GacA and RocAS two component systems, the RsmAYZ global post-transcriptional regulatory system, or the second messenger cyclic-di-GMP, all of which have been implicated in establishing chronic phenotypes in *P. aeruginosa* [66], [70], [71], [72], [73]. Studies on the molecular mechanisms underpinning the response of gastrointestinal bacteria to bile have recently led to the identification of a bile ‘sensor’ in *Listeria monocytogenes* [74], while a role for two component systems has also been reported in *S. enterica* [75]. Elucidating the molecular mechanisms underpinning the microbial response to bile, through these or alternative signalling pathways, will contribute significantly to our understanding of the factors underlying chronic respiratory infections.

In summary, GER-aspired bile may have a major influence on behaviour and biodiversity in the respiratory tract and lungs of CF and other patients (Fig. 5). In addition to eliciting chronic behaviour in a range of pathogens, aspired bile may also modulate community behaviour in the respiratory tract through the increased production of PQS and HHQ which were recently shown to modulate interspecies and interkingdom behaviour, including that of several CF pathogens [76]. The capacity for bile to trigger chronic behaviour in several CF pathogens, while reducing biofilm formation in *S. aureus* and *S. maltophilia*, may be highly significant in light of the epidemiology of CF infection where *S. aureus* is overtaken by *P. aeruginosa* as the dominant CF pathogen in patients >3 yrs of age [19]. However, it is worth noting that a single study performed by van der Doef and colleagues has reported earlier acquisition of *P. aeruginosa* and *S. aureus* in CF patients with GER [16]. This suggests that aspired bile may significantly influence behavioural traits other than biofilm formation in a range of respiratory pathogens. Therefore, a more comprehensive investigation into the extent of the influence of bile on these important CF pathogens needs to be undertaken to begin to understand the role of bile aspiration in controlling community behaviour in the pulmonary system. Ultimately it is hoped that this will provide a platform for designing innovative and effective strategies to treat what may be a major underlying cause of chronic respiratory disease.

## Supporting Information

Figure S1 **Bile concentrations as low as 0.03% modulate respiratory pathogen behaviour.** (**A**) Biofilm formation in multiwell plates was altered in the presence of 0.03% bile in a broad spectrum of respiratory pathogens. Bioflm was measured through crystal violet staining of attached cells and subsequent spectrophotometric analysis (Abs_595 nm_) in ethanol. Biofilm formation was enhanced in *A. baumannii*, *P. spuronum*, *P. aeruginosa*, and *B. cepacia*, while biofilm was reduced in *S. maltophilia* and *S. aureus*. (**B**) Swarming motility was reduced in the presence of 0.03% bile in wild-type and clinical isolates of *P. aeruginosa*. The swarm front distance was measured from the point of inoculation and relative distances calculated between control and treated plates. (**C**) Promoter activity of the T3SS effector *exoU* was significantly reduced in the presence of 0.03% bile. A reduction in promoter activity of 3-fold (+/−0.76 SEM) was observed in PA14 carrying a chromosomally inserted pMini-CTX-*exoU* promoter fusion. All experiments were performed in triplicate and data presented is representative of at least three independent biological replicates (p<0.005).
(PDF)

Figure S2 **Bile (0.03%) increases expression of** ***P. aeruginosa*** **QS systems in a dose dependent manner.** Addition of bile concentrations as low as 0.03% was sufficient to trigger expression of the PQS, Rhl and Las QS systems in *P. aeruginosa*. Promoter activity was significantly increased for all three systems in the presence of bile. The effect on *pqsA*, *rhlI*, and *lasI* promoter activity was more pronounced in the presence of 0.3% bile indicating that, as with other bile mediated phenotypes such as swarming and biofilm, the effect on QS is dose dependent. All experiments were performed in triplicate and data presented is representative of at least three independent biological replicates (*p<0.05, ***p<0.005).
(PDF)
