# Supplementary figures and images for: Respiratory Pathogens Adopt a Chronic Lifestyle in Response to Bile
Source: PLoS One. 2012 Sep 26;7(9):e45978. doi: 10.1371/journal.pone.0045978 (PMC3458808; doi:10.1371/journal.pone.0045978)

A

## Biofilm/Attachment Assay

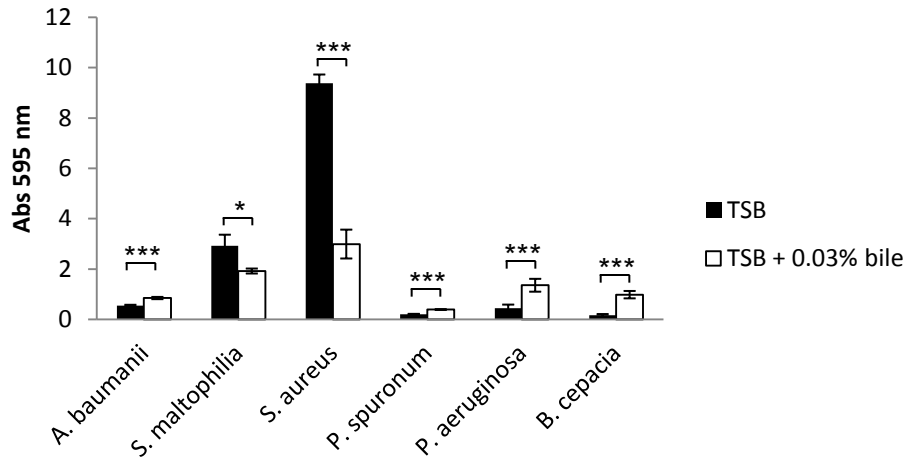

B

## Swarming Motility

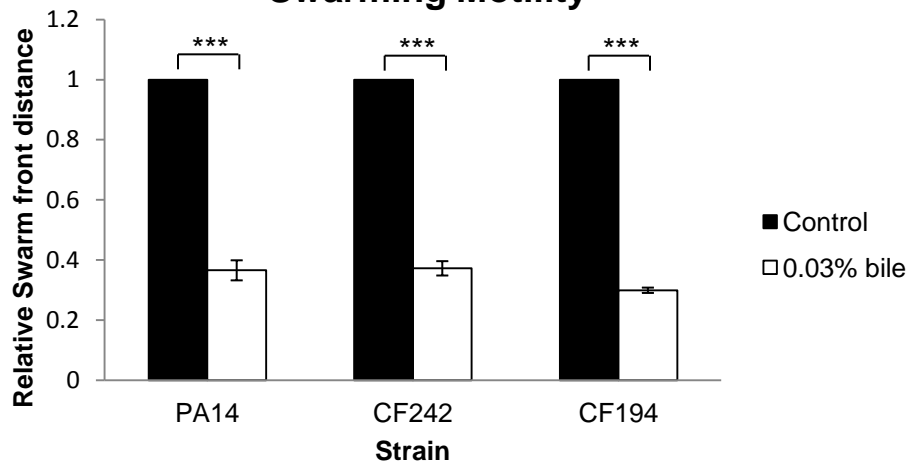

C

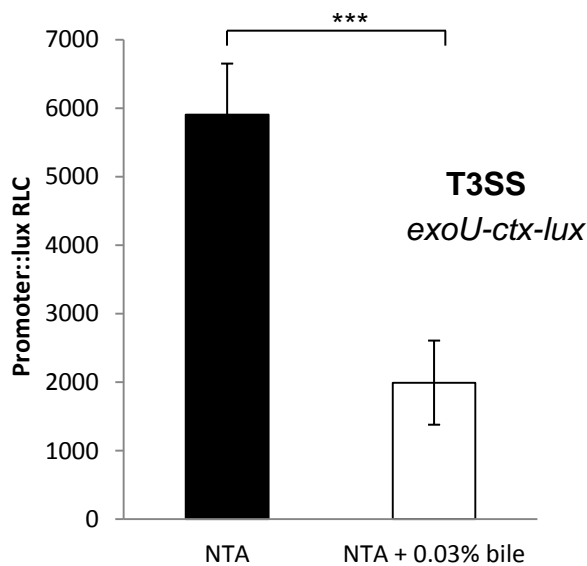

Supplement: Figure S1 — Bile concentrations as low as 0.03% modulate respiratory pathogen behaviour. (A) Biofilm formation in multiwell plates was altered in the presence of 0.03% bile in a broad spectrum of respiratory pathogens. Bioflm was measured through crystal violet staining of attached cells and subsequent spectrophotometric analysis (Abs595 nm) in ethanol. Biofilm formation was enhanced in A. baumannii, P. spuronum, P. aeruginosa, and B. cepacia, while biofilm was reduced in S. maltophilia and S. aureus. (B) Swarming motility was reduced in the presence of 0.03% bile in wild-type and clinical isolates of P. aeruginosa. The swarm front distance was measured from the point of inoculation and relative distances calculated between control and treated plates. (C) Promoter activity of the T3SS effector exoU was significantly reduced in the presence of 0.03% bile. A reduction in promoter activity of 3-fold (+/−0.76 SEM) was observed in PA14 carrying a chromosomally inserted pMini-CTX-exoU promoter fusion. All experiments were performed in triplicate and data presented is representative of at least three independent biological replicates (p<0.005). (PDF) [file pone.0045978.s001.pdf]

(i)

*pqsA-lacZ* pLP0996

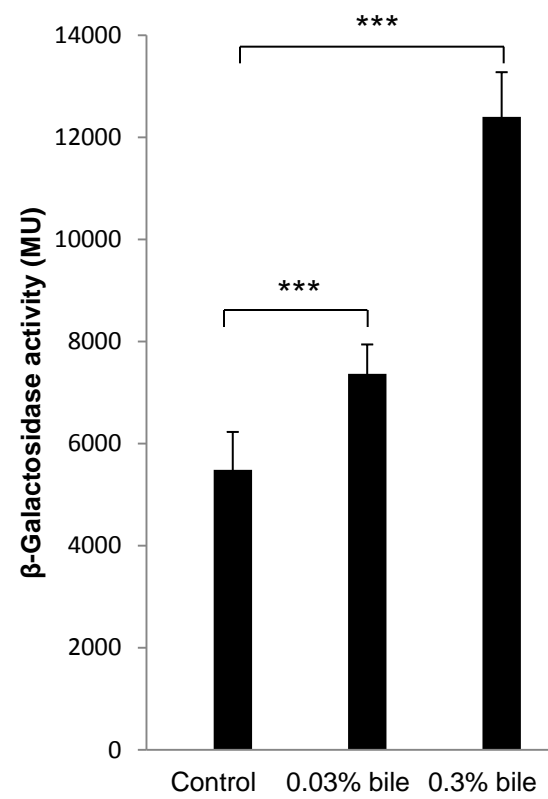

(ii)

*rhII-lacZ* pMP220

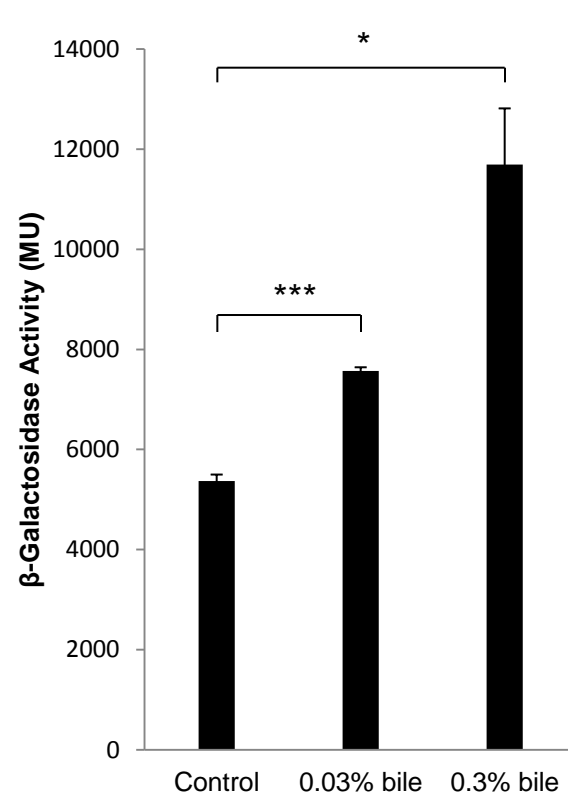

(iii)

*lasI-lacZ* pMP220

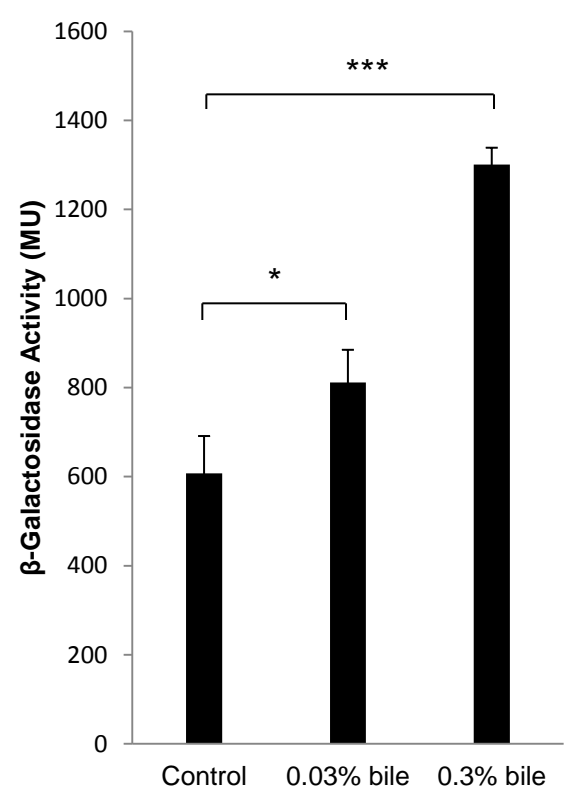

Supplement: Figure S2 — Bile (0.03%) increases expression of P. aeruginosa QS systems in a dose dependent manner. Addition of bile concentrations as low as 0.03% was sufficient to trigger expression of the PQS, Rhl and Las QS systems in P. aeruginosa. Promoter activity was significantly increased for all three systems in the presence of bile. The effect on pqsA, rhlI, and lasI promoter activity was more pronounced in the presence of 0.3% bile indicating that, as with other bile mediated phenotypes such as swarming and biofilm, the effect on QS is dose dependent. All experiments were performed in triplicate and data presented is representative of at least three independent biological replicates (*p<0.05, ***p<0.005). (PDF) [file pone.0045978.s002.pdf]
